# A DIY Fabrication Approach of Stretchable Sensors Using Carbon Nano Tube Powder for Wearable Device

**DOI:** 10.3389/frobt.2021.773056

**Published:** 2021-11-11

**Authors:** Ardi Wiranata, Yunosuke Ohsugi, Ayato Minaminosono, Zebing Mao, Haruyuki Kurata, Naoki Hosoya, Shingo Maeda

**Affiliations:** ^1^ Smart Materials Laboratory, Department of Engineering Science and Mechanics, Shibaura Institute of Technology, Tokyo, Japan; ^2^ Department of Mechanical and Industrial Engineering, Universitas Gadjah Mada, Yogyakarta, Indonesia; ^3^ Department of Engineering Science and Mechanics Shibaura Institute of Technology, Tokyo, Japan

**Keywords:** DIY, electroactive polymers, powder-based electrode, stretchable sensor, wearable sensor

## Abstract

Soft robotics and wearable devices are promising technologies due to their flexibility. As human-soft robot interaction technologies advance, the interest in stretchable sensor devices has increased. Currently, the main challenge in developing stretchable sensors is preparing high-quality sensors *via* a simple and cost-effective method. This study introduces the do-it-yourself (DIY)-approach to fabricate a carbon nanotube (CNT) powder-based stretchable sensor. The fabrication strategy utilizes an automatic brushing machine to pattern CNT powder on the elastomer. The elastomer ingredients are optimized to increase the elastomer compatibility with the brushing method. We found that polydimethylsiloxane-polyethyleneimine (PDMS-PEIE) is 50% more stretchable and 63% stickier than previously reported PDMS 30-1. With these improved elastomer characteristics, PDMS-PEIE/multiwalled CNT (PDMS-PEIE/MWCNT-1) strain sensor can realize a gauge factor of 6.2–8.2 and a responsivity up to 25 ms. To enhance the compatibility of the powder-based stretchable sensor for a wearable device, the sensor is laminated using a thin Ecoflex membrane. Additionally, system integration of the stretchable sensors are demonstrated by embedding it into a cotton-glove and a microcontroller to control a virtual hand. This cost-effective DIY-approach are expected to greatly contribute to the development of wearable devices since the technology is simple, economical, and reliable.

## Introduction

Soft robotics and soft wearable sensors are receiving increased attention due to their potential applications such as for rehabilitation or assistance purposes (Wehner et al., 2013; Polygerinos et al., 2015), human health monitoring systems (Ramasamy and Balan, 2018; He et al., 2021), human-machine interactions (Zoss et al., 2006; Banala et al., 2008; Roh et al., 2015), and human motion monitoring (Yang et al., 2018). Many soft actuator devices have already been developed. Examples of soft actuators include soft pneumatic actuators (Faudzi et al., 2018; Mohamed et al., 2020; Tschiersky et al., 2020; Ying et al., 2020; Luo et al., 2021), soft electroadhesion (Shintake et al., 2016; Guo et al., 2018; Okuno et al., 2019; Persson and Guo, 2019), stretchable pumps (Cacucciolo et al., 2019; Seki et al., 2020; Shigemune et al., 2021), dielectric elastomer actuators (DEAs) (Ji et al., 2019; Kajiwara et al., 2019; Minaminosono et al., 2019; Wang et al., 2019; Hiruta et al., 2021; Takagi et al., 2021; Wiranata et al., 2021; Zhao et al., 2021) a shape memory polymer (Besse et al., 2017; Li et al., 2020; Miao et al., 2020; Scalet, 2020; Zeng et al., 2020), and gel actuators (Maeda et al., 2007, 2010; Hwang et al., 2019; Mao et al., 2020a, 2020b; Zhang et al., 2020). To create an interface between soft actuators and human activity, the actuator must be equipped with an appropriate sensing method. One example is a pneumatic exoskeleton (Polygerinos et al., 2015), where the exoskeleton amplifies the normal muscle work precisely when the sensor and actuator are fully calibrated with the human motion.

In the context of human motion detection and soft robotics actuation monitoring, strain sensing is essential to detect deformation of the system. Although conventional strain sensors can convert the strain produced by the external stimulation into a signal response (Han et al., 2017), they cannot detect the full range of human motions. The conventional strain sensor structure usually consists of a high stiffness material conductor or semiconductor, limiting the strain range sensing (Barlian et al., 2009). Since the movement of human joints induces a large strain range (more than 30%), flexible strain sensors with a high stretchability are needed to detect the full range of human motions.

In the sensor-actuator integrated system, the flexible strain sensor provides feedback to the system by sending the resistance data change. The change in electrical resistance *R* of the flexible strain sensor is affected by the strain *ε* of the elastomer. The relative change of *R* to the *ε* is denoted as the gauge factor (GF) which also known as the sensitivity of the sensor. When the flexible sensor undergoes a uniaxial stress, the sensor should expand in one direction causing a change in the geometric shapes. The change in geometric shape is described as follows in Equation 1 (Shintake et al., 2018).

$$l_{e}=\varepsilon_{1}l_{e0}+l_{e0},w_{e}=\frac{w_{e0}}{\sqrt{\varepsilon_{1}+1}},t_{e}=\frac{t_{e0}}{\sqrt{\varepsilon_{1}+1}},$$ (1)

Where *l*_*e*_ is the electrode length, *w*_*e*_ electrode width, *t*_*e*_ electrode thickness and *ε*_*1*_ is the strain in the loading direction. *l*_*e0*_, *w*_*e0*_, *t*_*e0*_, are initial length, width, and thickness of the electrodes respectively. By assuming the cross section of the flexible sensor is uniform, the resistance change can be described as follow (Eq. 2) (Shintake et al., 2018)

$$R=\rho \frac{l_{e0}}{w_{e0}t_{e0}}{\left(\varepsilon_{1}+1\right)}^{2}=\frac{\rho }{\rho_{0}}R_{0}{\left(\varepsilon_{1}+1\right)}^{2},$$ (2)

Where *ρ*_*0*_ is the reference resistivity, *ρ* is the resistivity of stretchable electrodes, and *R*_*0*_ is the reference resistance. Then the sensitivity of the sensor (GF) can be defined using Eq. 3 (Shintake et al., 2018)

$$\mathrm{GF}=\frac{\Delta R}{R_{0}\varepsilon_{1}}=\frac{1}{\varepsilon_{1}}\left(\frac{\rho }{\rho_{0}}{\left(\varepsilon_{1}+1\right)}^{2}-1\right).$$ (3)

Many researchers have focused on producing high-quality stretchable strain sensors. To improve the sensor quality (in terms of sensitivity, stretchability, and reliability), both material selection and the fabrication method are necessary to optimize devices containing stretchable electrodes. Different types of stretchable electrodes have been investigated, including carbon-based electrodes (Rosset and Shea, 2013), metal thin-film electrodes (Rosset and Shea, 2013), composite silicone-carbon electrodes (Shintake et al., 2018), and ionic gels (Keplinger et al., 2013). Each electrode has distinctive characteristics when it is used as a stretchable strain sensor.

Several fabrication methods have been reported for strain sensors, including a lamination technique for stretchable electrodes, mixing and creating electrodes-polymer composites, and direct electrode patterning on the elastomer. There are some examples of lamination techniques such as electrode pad printing (Poulin et al., 2015), Langmuir-Schaefer (LS) (Ji et al., 2018), and supersonic cluster beam implantation (SCBI) (Taccola et al., 2018). Among these methods, pad printing is the simplest to create stretchable electrodes because user can stamp the electrodes directly on the elastomer surface using a commercial pad-printing machine. Pad printing requires a liquid type of electrode, which requires additional treatment [e.g., mixing of carbon black and elastomer to form a carbon black-elastomer composite (Poulin et al., 2015)] to produce high-quality stretchable electrodes. Moreover, this methods also require a pad-printing machine (Poulin et al., 2015) which can add additional cost to the overall production of stretchable electrodes. In the case of SCBI (Taccola et al., 2018), it requires special equipment that can accommodate high pressure. The SCBI process also requires an additional inert gas which adding an additional cost to the fabrication process. In the case of LS (Ji et al., 2018), the method is simply by transferring the electrodes from the surface of the water to the target surface (e.g., PDMS surface), this method requires a good combination of conductive material before transferring to the target surface [e.g., a composite between hydrophobic poly (alkylthiophene) and hydrophilic multiwalled carbon nanotubes (MWCNT) (Ji et al., 2018)]. The material addition also can cause an additional cost. Moreover, a special cleanroom is also needed since the working properties is liquid and to avoid any contaminations.

Another fabrication technique is to mix and create electrode-polymer composites (Shintake et al., 2018; Teixeira et al., 2018; Jin et al., 2020; Dong et al., 2021; Song et al., 2021). This popular method can create stretchable electrodes for strain sensors but a long mixing process is required to realize a uniform mixing solution. Another fabrication method is electrode patterning by 3D printing (Muth et al., 2014). In this process, the key is to produce a good substrate quality before the electrodes are printed on the elastomer. Hence, substrate preparation requires additional complicated processes and special skills to achieve a good substrate composition.

Currently, the development of stretchable strain sensors *via* rapid, easy, reliable, and cost-effective fabrication methods remains challenging although the demand for stretchable strain sensor products is high (Song et al., 2021). In the soft robotics area, there are several fast fabrication techniques known as do-it-yourself (DIY) methods. In principle, DIY is an activity where researchers create a product or a process through individual or a collective production practice for a specific personal purpose, and usually the methods used are by researcher’s own innovation (Wolf and Mcquitty, 2011; Camburn and Wood, 2018). The DIY in soft robotics area can produce highly reliable actuators, including flexible electrohydrodynamic (EHD) pump technology (Seki et al., 2020) and basic dielectric elastomer actuator (DEA) technology (Wiranata et al., 2021). DIY methods are popular because they offer users the freedom to modify and define the parameters during device fabrication. Many open sources and communities exist to support the method. The DIY method also has been implemented in other areas such as biotechnology (Wong et al., 2018), biochemistry (Szymula et al., 2019), and other topics related to laboratory development (Kubínová and Šlégr, 2015; Grinias et al., 2016; Vallejo et al., 2020). Reliable DIY for rapid prototyping is essential to accelerate the development and innovation in soft robotics.

Previously, we have developed a method to reliably fabricate stretchable electrodes using the brushing method of CNT powder for DEAs (Minaminosono et al., 2021; Murakami et al., 2021; Wiranata et al., 2021; Wiranata and Maeda, 2021). Hand brushing CNT on the elastomer is a relatively easy and fast process. In order to eliminate the human influence in the brushing process, improve the consistency of the brushing and ease of brushing process, we automate the brushing process using brushing machine. An automatic brushing machine is a convenient technology for future mass production. For the brushing machine, we customized a low-cost commercial DIY-kit three-axis machine tools to realize an automatic brushing process. Then we improved the elastomer compatibility towards fabrication of stretchable strain sensor using automatic brushing method. The elastomer compatibility and quality was optimized by adding polyethyleneimine (PEIE) in the previously reported pre-polymer of polydimethylsiloxane **(**PDMS) 30-1 (Wiranata et al., 2021). Adding a small amount of PEIE (approximately 0.11 wt%) increased the stickiness of the elastomer, enhancing the compatibility for the brushing process of the CNT powder. To examine the effect of powder size on the stretchable strain sensor properties, we assessed three conductive powders: multiwalled carbon nanotubes (MWCNTs) with outer diameters of 6–9 nm (MWCNTs-1), MWCNTs with outer diameters of 10–20 nm (MWCNTs-2), and MWCNTs with outer diameters of 50–90 nm (MWCNTs-3).

Then to enhance the compatibility of the sensor for wearable devices, we introduced a lamination process using a thin Ecoflex membrane for the stretchable strain sensor after the brushing process. The Ecoflex membrane can cover the sensor since both the Ecoflex membrane and sensor display high stretchability mechanical characteristics. A high stretchability can ease material handling of micrometric layers of the Ecoflex membrane since the membrane does not rip easily during the lamination process. Finally, we integrated a stretchable strain sensor with a low-cost microcontroller (Arduino) as the signal processor to detect human hand movement. The hand movement was captured and displayed using virtual software.

Our study aims to provide a DIY approach for rapid, easy, reliable, and cost-effective fabrication methods for fully laminated stretchable strain sensors using automatic brushing machines to brush a carbon nanotube powder on the elastomer. The reasons for the cost and time reduction in our DIY approach include the use of low-cost commercial DIY-kit three-axis machine tools covered with a portable glovebox that can ease the brushing process in regular laboratory conditions. This can eliminate the requirement of a costly cleanroom. Furthermore, a direct brushing of CNT on the surface of the PDMS can speed up the fabrication process.

**FIGURE 1:**
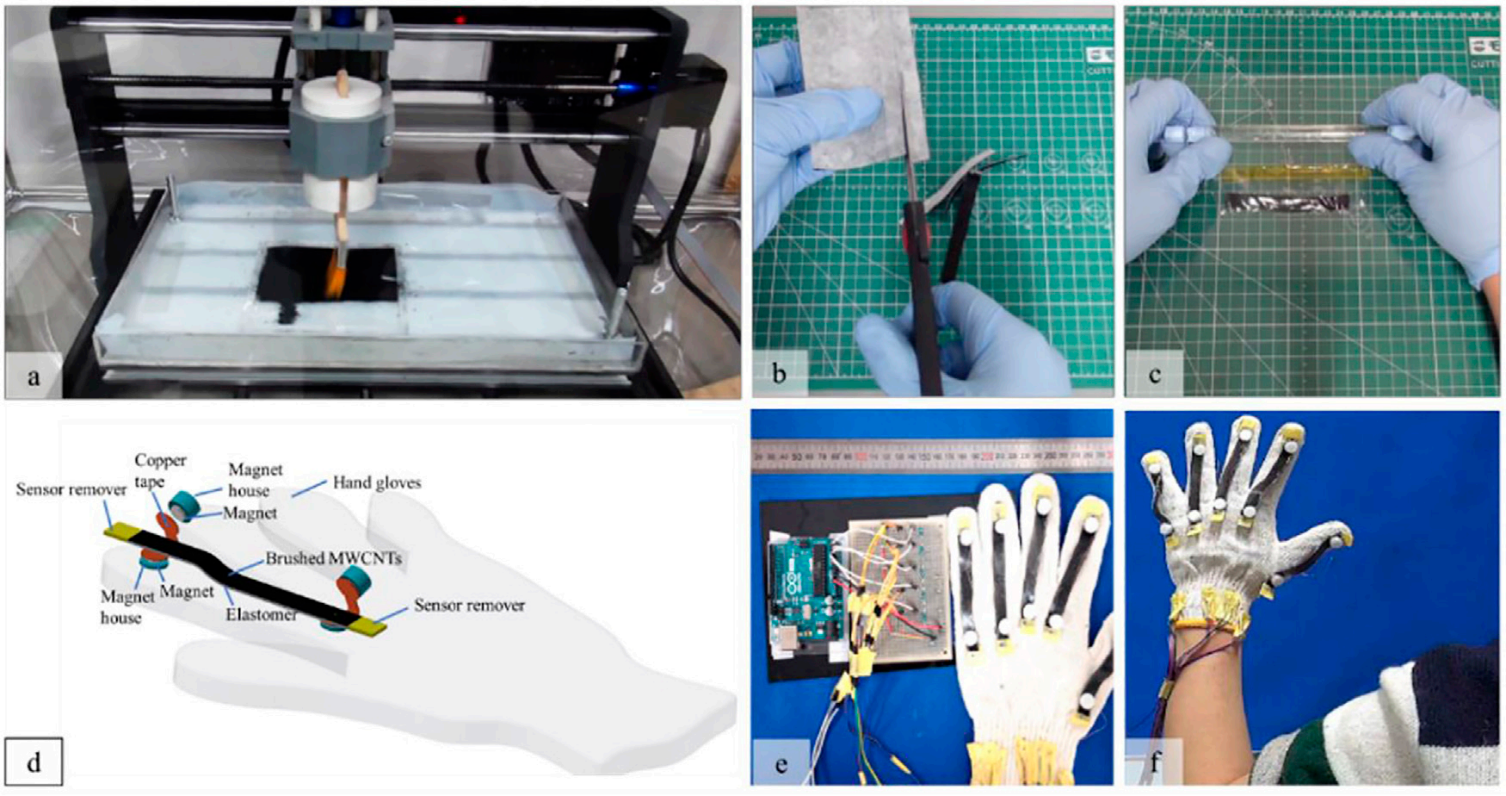
DIY**-**fabrication concept of a stretchable strain sensor and a wearable device prototype. **(A)** Brushing of the CNT powder using a brushing machine, **(B)** manually shaping of the stretchable sensor, and **(C)** laminating the stretchable strain sensor using thin Ecoflex membrane. **(D)** Plug-and-play concept for ease of sensor mounting and maintenance, **(E)** sensor embedment on a cotton glove and integration system using an economical microcontroller (Arduino) and **(F)** demonstration of the strain sensor to detect human hand movement.

As a prototype of our powder-based stretchable sensor for wearable device, we integrate the sensor with a low-cost microcontroller (Arduino) to detect hand movements. Figure 1 depicts the overall fabrication sequence of the powder-based stretchable sensor and the wearable device prototype. We expect that our novel DIY strategy will contribute to the development of human-soft robotics interactions. Many researchers can easily access this proposed technology since our fabrication process is simple and reliable. We believe this research can attract more researchers and lead to the faster development of soft robotics, especially the human-soft robotics interface.

## Materials and Methods

### Elastomer Fabrication

We used a commercially available elastomer, Sylgard 184 by Dow Inc., to produce a stretchable strain sensor. This PDMS comes in two liquid parts: the elastomer base and the curing agent. The standard recommended mixing ratio is 10 g of the elastomer base to 1 g of the curing agent. Both elastomer and curing agent can be mixed manually or using a commercial mixer (e.g., Thinky mixer AR-100) to ensure the uniformity of the mixing solution. The solution can be cured by either sitting at room temperature or baking in the oven to shorten the curing time. Previous reports have indicated that modifying the Sylgard 184 mixing ratio changes the mechanical characteristics of the elastomer (Kim et al., 2011; Johnston et al., 2014; Wiranata et al., 2021).

Since we used powder-type electrodes, the elastomer stickiness should be optimized to strengthen the physical bonding between the elastomer surface and the multiwalled carbon nanotube (MWCNT) powder. This strong bond reduces the risk of the MWCNT powder detaching from the elastomer surface. Reducing the curing agent part to 3.2 wt% can realize a sticky PDMS surface (Wiranata et al., 2021). To further improve the stickiness characteristics, we added PEIE (Yamamoto et al., 2017) (80% ethoxylated solution by Sigma-Aldrich) approximately of 0.11 wt% to the PDMS prepolymer with the 3.2 wt% of curing agent. Then the liquid solution was pre-mixed manually. We placed the pre-mixed solution into a commercial mixer (Thinky mixer AR-100) for 3 min at a speed of 2000 rpm to ensure a uniform mixture. We then let the PDMS cure at a temperature of 60°C for 4 h.

To evaluate the mechanical characteristics of the elastomer, we molded the PDMS-PEIE to create a sheet with the size of 145 × 135 × 2 mm^3^. After elastomer was fully cured, we prepared a specimen for a tensile test using a precision dumbbell blade (the size of the dumbbell blade is in accordance to JIS K6251). Then the specimen was tested using Shimadzu AGS-X with a loading speed of 500 mm/min for the uniaxial tensile test. All of the tensile test data were recorded automatically. For each condition, at least three samples were investigated.

### Sensor Fabrication

The PDMS-PEIE sheet was prepared using the same methods as mentioned in the previous section. We employed a simple coating process to create an approximately 0.5-mm-thick elastomer membrane. After fully curing, the elastomeric membrane was peeled from the acrylic plate. We then attached it to a 100 × 100 mm^2^ acrylic frame. An acrylic frame can ease material handling when a brushing method is applied.

The fabrication of PDMS-powder-based stretchable strain sensor begins with brushing process for the CNT powder on the PDMS-PEIE. We applied a DIY approach by utilizing an automatic brushing machine. The automatic brushing machine was a customized commercially available DIY-Kit x-y-z machine tool (SainSmart Genmitsu CNC Router 3018-PRO). To ease the CNT powder handling in the brushing process, we put the x-y-z machine tool inside a commercially available glove box (As One 3-116-01 SM-1). Additionally, the brush was also a readily available commercial paintbrush with nylon hair (Supplementary Figure S1).

**FIGURE 2:**
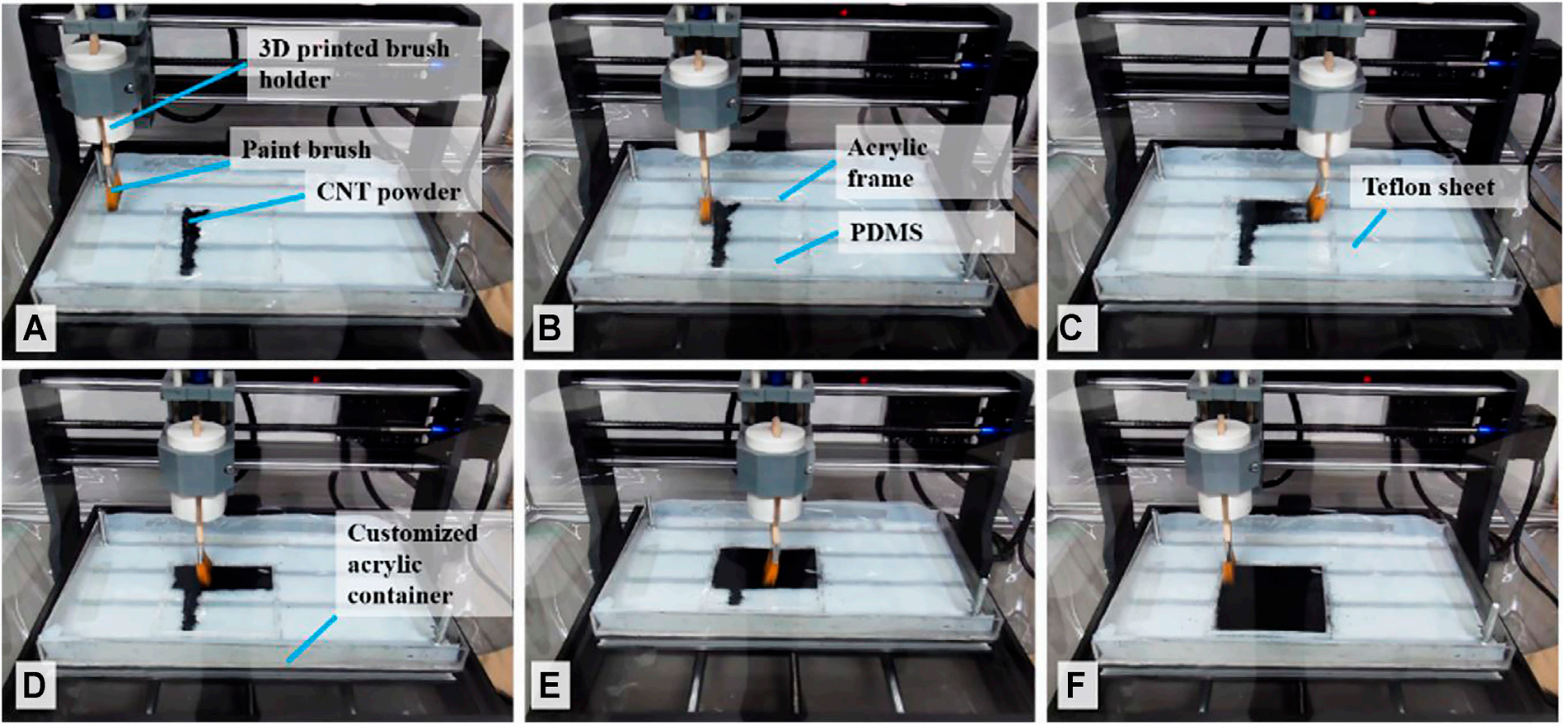
Equipment setup and brushing process using a brushing machine. (See Supplementary Movie S1 to view the complete process).

**FIGURE 3:**
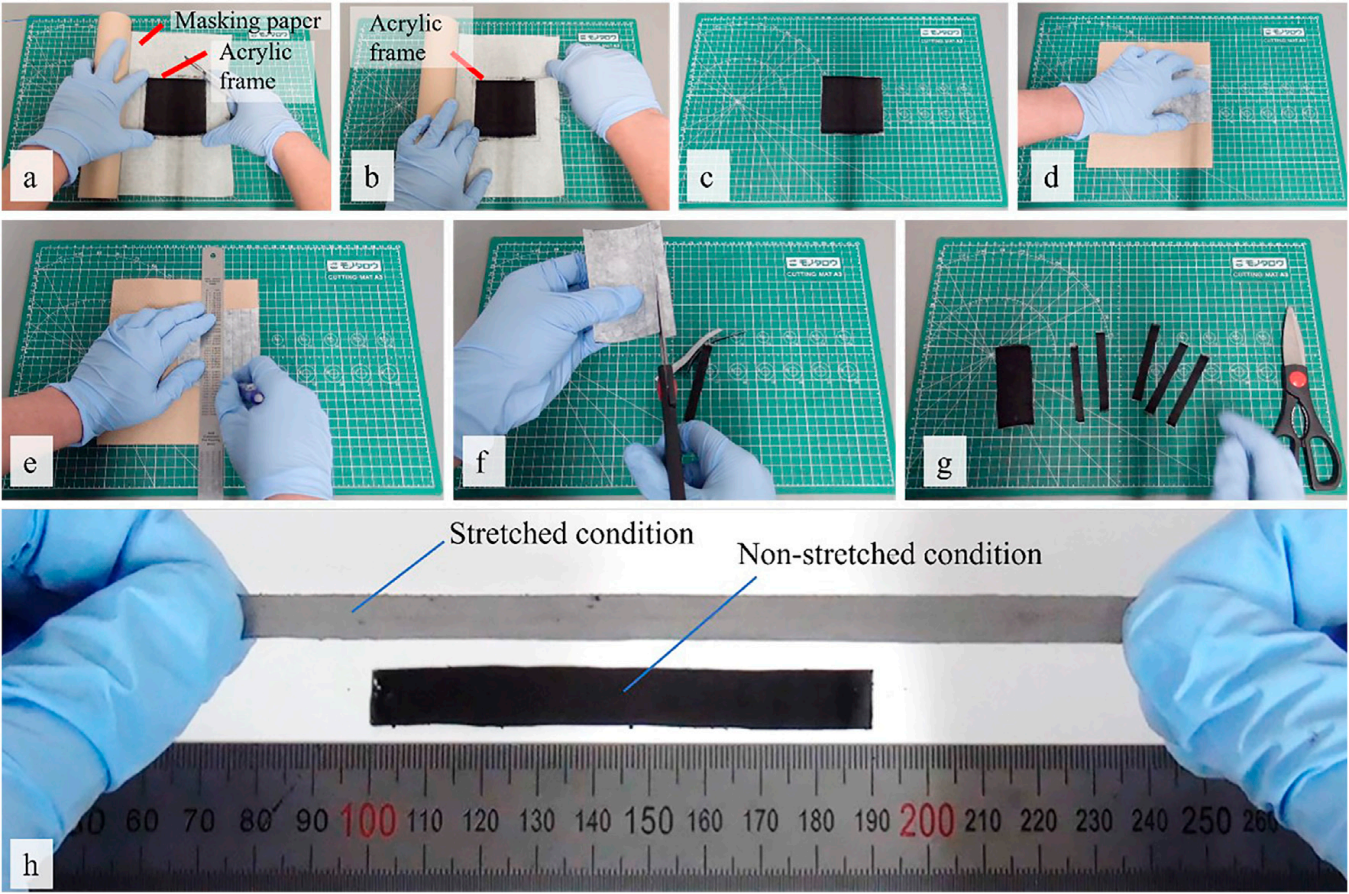
Shaping process of the stretchable strain sensor.

The brushing process began by placing the electrode powder on the elastomer surface (Figure 2A). We pre-poured approximately 0.02 g of MWCNTs powder from the top left corner to the bottom left of the PDMS-PEIE surface to form a line as shown in Figure 2A. Then a series of bi-directional planar sweeps along linearized rail brushings was performed at a speed of 1000 mm/min (Figures 2C,D, and 2E) until the entire elastomer surface was covered with the electrode powder (Figure 2F). To ensure a uniform brushing process, we performed 20 cycles of brushing for each position. Supplementary Movie S1 shows the details of the brushing process. After the brushing process, we shaped the brushed PDMS-PEIE into a 10-mm-wide strip by manually cutting the sheet (Figure 3). To ease the sensor shaping process, the brushed PDMS-PEIE with an acrylic frame was placed on the masking paper with the brushed surface facing upward prior to removing the acrylic frame (Figure 3A). Then we cut the PDMS-PEIE using a scalpel (Figure 3B) to form a square brushed PDMS-PEIE sheet without a frame (Figure 3C). The sheet was subsequently flipped backward and placed on a soft surface (e.g., tissue paper) (Figure 3D). We manually drew lines on the masking paper with a 10-mm gap between each line (Figure 3E). Finally, we cut the strip according to the pattern (Figure 3G). Figure 3G shows the final product of the stretchable strain sensor. We needed to peel the stretchable strain sensor from the masking paper. Figure 3H shows the stretchable strain sensor in unstretched and stretched states.

### Electromechanical Sensor Testing

**FIGURE 4:**
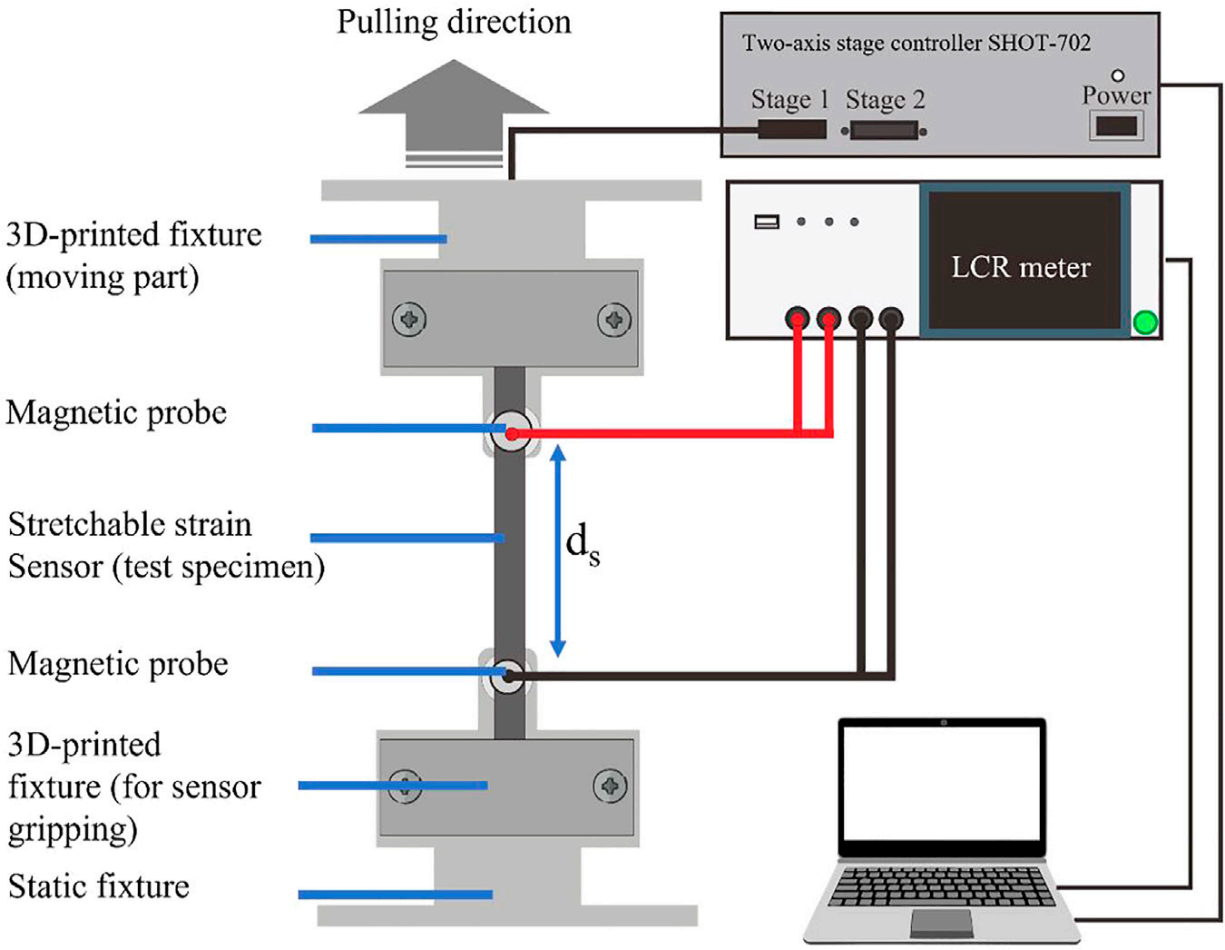
Electromechanical experimental setup for sensor characterization.

To investigate the characteristics and reliability of the stretchable strain sensors, we performed a series of cyclic electromechanical tests. We initially attached the sensor to a 3D printed fixture mounted on a linear motorized stage (SGSP26-200 by Sigmakoki) for cyclic uniaxial loading and unloading. The pulling speed and the displacement of the linear motorized stage were controlled using SHOT-702 (Sigmakoki). The resistance value of the sensor was measured using an LCR meter (IM3536 by Hioki) that was set to a sampling frequency of 200 Hz. The LCR meter data and the displacement data from the motorized stage were recorded using a personal computer. We used a recording speed of 20 Hz. The length between measurement probe (*d*_*s*_) was 70 mm. Figure 4 shows the detailed arrangement of the equipment. To evaluate the influence of the strain speed on the sensor sensitivity, the stretchable sensor was uniaxially pulled until a 40% strain at a constant speed of 7, 21, or 30 mm/s. Each strain speed was repeated for 50 cycles.

### Sensor Lamination Process

Wearable sensors are currently receiving much attention due to the growing need for a reliable human-machine interface. Herein, we demonstrate that our stretchable sensor fabricated using the DIY approach is suitable for a wearable sensor. We performed a lamination using a thin Ecoflex membrane to improve the compatibility of the sensor toward wearable device. The Ecoflex membrane was fabricated by spin-casting an Ecoflex solution at 2000 rpm for 30 s. Then, the spin-casted Ecoflex was cured at 60°C for 10 min. The thickness of the spin-casted Ecoflex membrane was evaluated after it was fully cured using a thickness screw gauge and was approximately 30–50 µm-thick. Since Ecoflex is a highly stretchable material, the thin Ecoflex membrane can easily be removed from the casting substrate.

**FIGURE 5:**
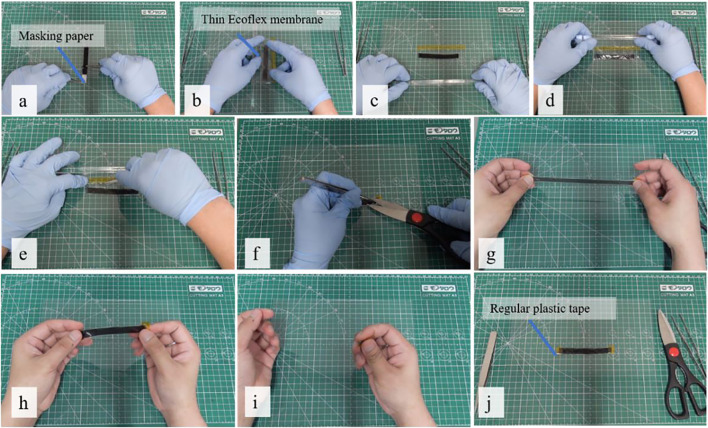
Sensor lamination process using a thin Ecoflex membrane.

Figure 5 shows the lamination process. First, the sensor was peeled from the masking paper (Figure 5A). Then the stretchable sensor was fully covered with a thin Ecoflex membrane (Figures 5B–D). Afterward, the unnecessary parts were removed using a cutter or scalpel (Figure 5E). Then the final step was to remove a small part of the Ecoflex membrane to create a contact surface (Figure 5F). We tested the lamination quality by manually stretching (Figure 5G) and squeezing (Figure 5I) the sensor. Figures 5H,J demonstrate that squeezing and stretching do not induce a noticeable delamination of the Ecoflex thin membrane. (Supplementary Figure S2 shows the final structure of the sensor.) We did not use any adhesive to apply the thin membrane to the stretchable sensor since the PDMS-PEIE already has a sticky nature characteristic. After the lamination process, we investigated the characteristics of the laminated sensor using the same equipment shown in Figure 4.

**FIGURE 6:**
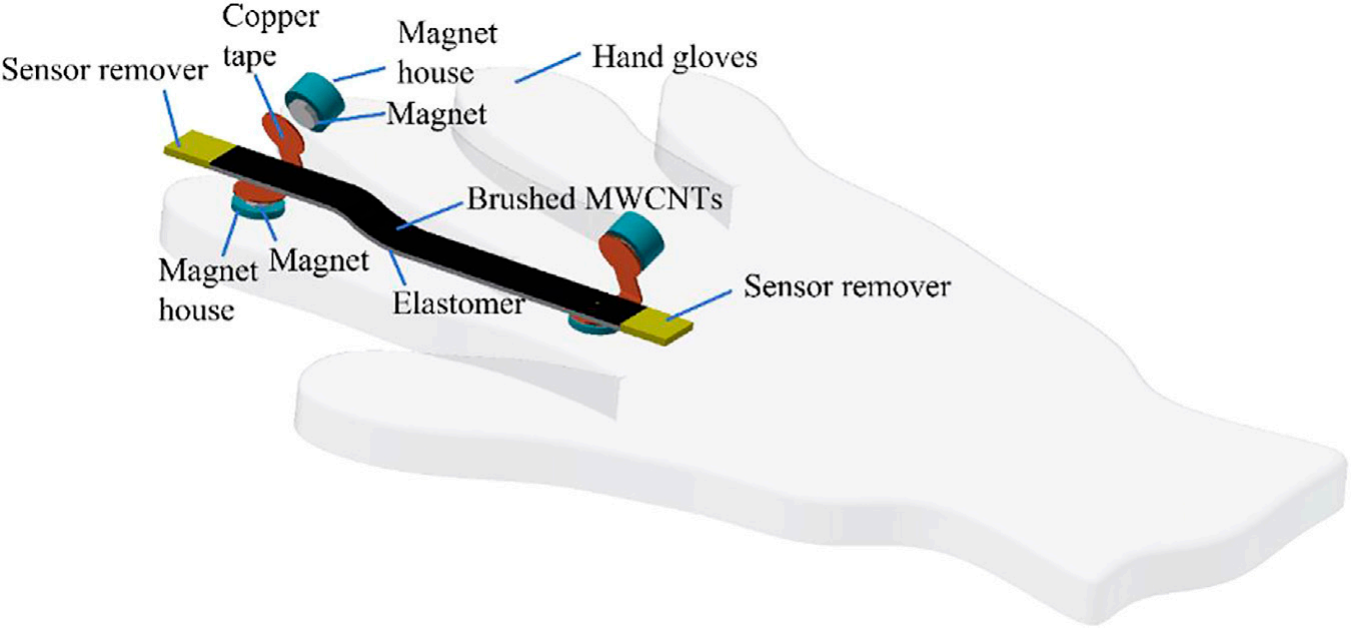
Plug-and-play concept of the stretchable strain sensor.

To simply attach the stretchable strain sensor to any object, including the human body or other wearable devices such as gloves or sleeves, we proposed a plug-and-play concept for this stretchable strain sensor (Figure 6). This plug-and-play concept allows the stretchable sensor to be easily placed and adjusted. For long-term sensor operations, this concept can minimize regular maintenance. If the sensor is damaged, it can easily be removed and replaced with a new one. Figure 6 shows an example where the device is attached to a glove. Additionally, the customized stretchable strain sensor mounting may help adjust the tension level of the stretchable strain sensor.

## Results

### Elastomer Mechanical Properties

**FIGURE 7:**
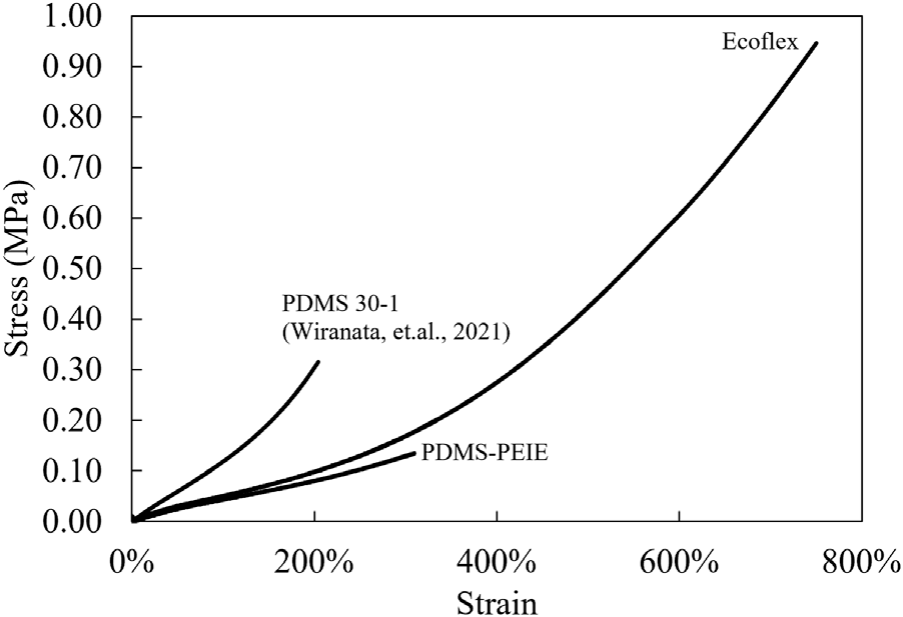
Mechanical characteristics (engineering stress-strain) of the elastomer.

Figure 7 shows comparison of the mechanical characteristics of PDMS-PEIE, PDMS 30-1, and Ecoflex. Adding PEIE to the PDMS changes the mechanical characteristics of the elastomer due to the crosslinking characteristics formed on the elastomer (Jeong et al., 2016). The addition of the PEIE in the elastomer solution realizes an approximately 50% (PDMS-PEIE) more stretchable elastomer compared to the previously reported PDMS with a 3.2 wt% curing agent and without PEIE (PDMS 30-1) (Wiranata et al., 2021). This improved maximum failure strain of the PDMS-PEIE is attributed to the heterogeneously crosslinked elastomer network (Jeong et al., 2016).

A tacky elastomer surface is necessary when a powder-type electrode is used. Additionally, an adaptable and viscous surface increases the physical bonding between powder-type electrodes and the elastomer surface. We employed the procedure from the American Society for Testing and Material (ASTM D 6195 03) for the loop tack test to assess the stickiness of the elastomer surface. We created a 0.5 × 25 × 175-mm^3^ PDMS strip using a simple molding method (Supplementary Figure S3 describes the molding shape and method). We then bent the specimen upon itself to form a pendant-drop shape loop. The ends of the loop were connected using masking tape. The loop was gripped by the tensile tester. Then the pendant drop shape was brought into contact with the polished stainless-steel surface to form a 25 × 25-mm^2^ contact area. Finally, the tensile tester pulled the test specimen with a speed of 300 mm/min until the elastomer fully detached from the stainless-steel surface. Details of the tackiness test are previously reported elsewhere by Wiranata et al. (2021).

**FIGURE 8:**
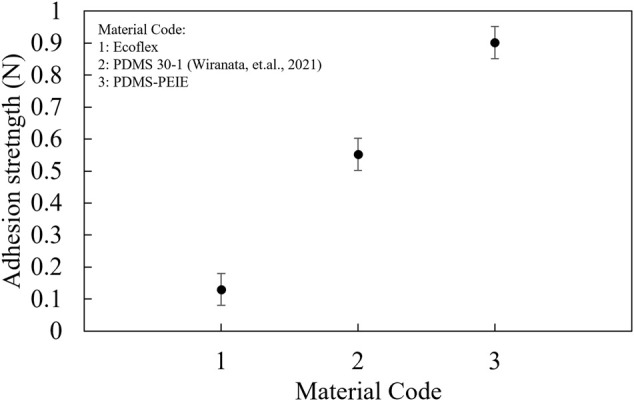
Tackiness of the elastomer surface.

Figure 8 shows the maximum adhesion strength of the elastomer. Adding a small amount of PEIE into the mixing solution of PDMS improves the elastomer stickiness. Compared to Ecoflex and previously optimized PDMS 30-1 (Wiranata et al., 2021), PDMS with PEIE has a higher adhesive strength. PDMS-PEIE has the highest adhesive strength since we added more PEIE (approximately 0.11 wt%) to the PDMS-PEIE. Adding a small amount of PEIE (approximately 0.11 wt%) realizes PDMS with an approximately 63% higher tackiness than the previously developed PDMS30-1 (Wiranata et al., 2021). The high stickiness characteristics of this elastomer possibly due to the viscous surface adaptation of the PDMS, which allows van der Waals interactions with another substrate surface (Jeong et al., 2016). Moreover, the surface adaptability of the elastomer enhances the surface contact area between the elastomer and substrate which further strengthening the bonding between the elastomer surface and the substrate.

### Surface Condition of the Brushed CNT on the Elastomer

To investigate the effect of CNT powder type on the stretchable strain sensor performance, we examined three different sizes of multiwalled CNTs: MWCNTs 724769-25G from Sigma-Aldrich with an outer diameter of 6–9 nm and 5 µm length (MWCNTs-1), MWCNTs 0553CA from Sky Spring Nanomaterial with an outer diameter of 10–20 nm and 5–30 µm length (MWCNTs-2), and MWCNTs 901019-25G from Sigma-Aldrich with an outer diameter of 50–90 nm and 15 µm (MWCNTs-3).

**FIGURE 9:**
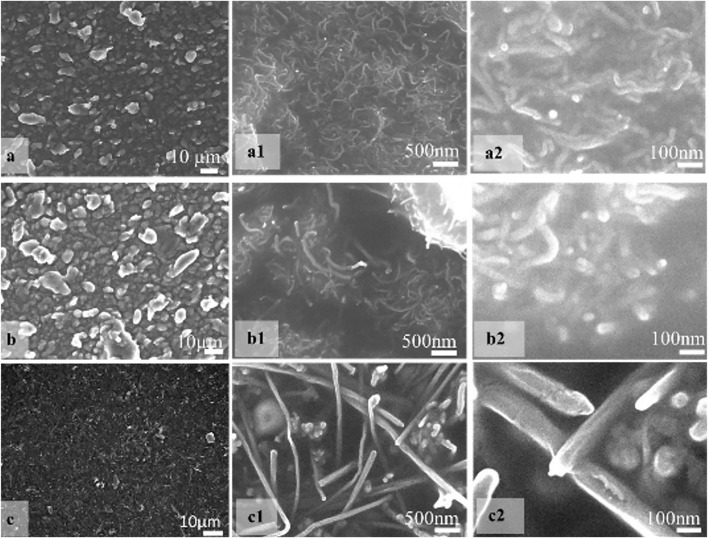
FE-SEM images show the surface morphology of **(A−A2)** PDMS-PEIE/MWCNTs-1, **(B−B2)** PDMS-PEIE/MWCNTs-2, and **(C−C2)** PDMS-PEIE/MWCNTs-3 at different magnifications (micrometric to nanometric size).

The surface of the brushed PDMS-PEIE was investigated using field-emission scanning electron microscopy (FE-SEM JSM-7610 F (JEOL)) to characterize the surface morphology of PDMS-PEIE brushed with different sizes of CNT. Figures 9A–A2 show the surface condition of PDMS-PEIE/MWCNTs-1. Agglomerates of several MWCNTs appear on the micrometric scale (Figure 9A). Compared to previously reported research on manual brushing of MWCNTs for dielectric elastomer actuators (Wiranata et al., 2021), the agglomerates of MWCNTs-1 in micrometric size (Figure 9A) shows more uniform size than previously reported research (Wiranata et al., 2021). This uniform size of the agglomerates is possibly due to the more consistent brushing (e.g., brushing pressure and brushing speed) in the brushing machine than in the manual brushing process. The agglomerates can affect the quality of the sensor. We expect that smaller agglomerates produce less noise in the sensor reading when the sensor is at a higher strain (e.g., 100% strain). Magnified images on the nanometric scale show that the MWCNTs-1 are spread uniformly on the surface of the PDMS-PEIE (Figures 9A, A2). The nanometric scale image in Figure 9A1 also shows that the MWCNTs network is spread more uniformly than the previously reported research on the manual brushing methods (Wiranata et al., 2021). This uniform dispersion pattern should enhance the network connection between MWCNTs particles.

A similar pattern is also observed in MWCNTs-3, which has a larger particle size (Figures 9C1, C2). This uniform pattern on the nanometric and micrometric scales (Figure 9C) lowers the noise reading of the strain sensor. Furthermore, the structure of MWCNTs-3 (Fig, 9c1) looks stiffer than the other MWCNTs. Figure 9C1 indicates a less dense MWCNTs network structure. This less dense network structure causes a problem when the strain sensor is under high strain conditions (e.g., degradation of the sensor sensitivity). In the case of PDMS-PEIE/MWCNTs-2, Figures 9B1, B2 show black blank spaces and other areas full of MWCNTs network, which resemble the valleys and peaks on the surface of PDMS-PEIE/MWCNTs-2 respectively. This surface pattern of PDMS-PEIE/MWCNTs-2 causes the strain sensor to have a higher sensitivity since the valleys and peaks can shift easily and collide with each other when the sensor size changes due to the strain effect. Compared to the PDMS-PEIE/MWCNTs-1 surface (Figure 9A), the PDMS-PEIE/MWCNTs-2 surface (Figure 9B) has bigger agglomerates, which can stimulate noise in the reading when the strain sensor operates at a higher strain.

### Electromechanical Properties of the Sensor

**FIGURE 10:**
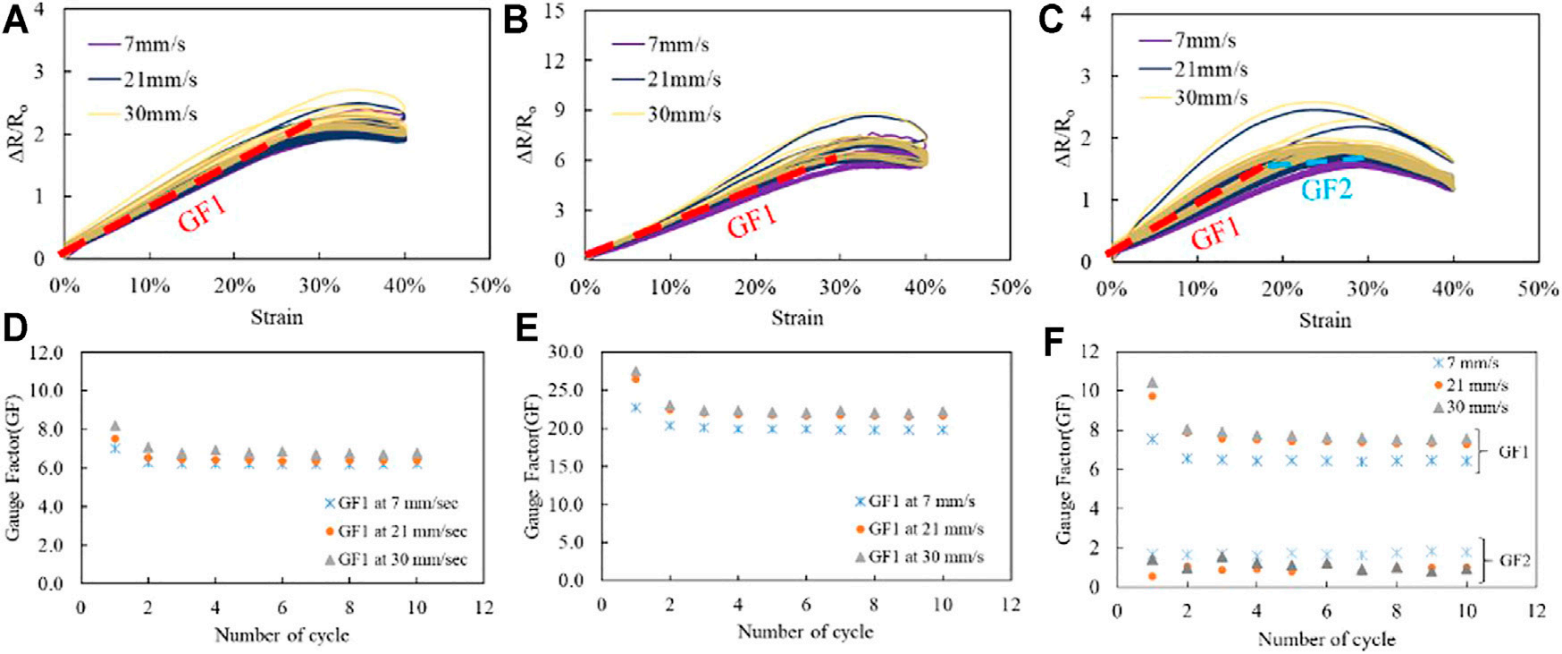
Electromechanical characteristics of the stretchable strain sensors: **(A)** PDMS-PEIE/MWCNTs-1, **(B)** PDMS-PEIE/MWCNTs-2 and **(C)** PDMS-PEIE/MWCNTs-3. Gauge factors (GFs) of **(D)** PDMS-PEIE/MWCNTs-1, **(E)** PDMS-PEIE/MWCNTs-2, and **(F)** PDMS-PEIE/MWCNTs-3.

Figure 10 describes the characteristics of the stretchable strain sensors fabricated using PDMS-PEIE. We tested three different types of MWCNTs. As expected, the sensitivity of the stretchable strain sensor does not change significantly after 50 stretching-relaxing cycles. The stretchable strain sensor reading converges from the 2nd cycle to the 50th cycle (Figures 10A–C). PDMS-PEIE/MWCNTs-1 has the most stable readings at different tensile speeds (Figure 10A). The gauge factor (GF) of the PDMS-PEIE/MWCNTs-1 stretchable strain sensor remains nearly constant even as the pulling speed increases (Figure 10D) The PDMS-PEIE/MWCNTs-1 stretchable sensor shows less noise than the PDMS-PEIE/MWCNTs-2 stretchable strain sensor at a tensile speed of 7 mm/s (Figure 10A). (Supplementary Figure S4 provides more details about the PDMS-PEIE/MWCNTs-2 stretchable sensor at a tensile speed of 7 mm/s). The PDMS-PEIE/MWCNTs-1 stretchable sensor can be stretched up to 100% strain, and the reading result shows less noise (Supplementary Figure S5A) than PDMS-PEIE/MWCNTs-2.

PDMS-PEIE/MWCNTs-2 (Figure 10B) shows a higher GF (Figure 10E) compared to the other stretchable strain sensors. The GF is also sensitive to the tensile speed (Figure 10E). PDMS-PEIE/MWCNTs-2 also has higher noise when it is stretched up to 100% strain (Supplementary Figure S5B). For the PDMS-PEIE/MWCNTs-3, the GF changes as the strain increase (Figure 10C). A smaller GF value appears at a strain above 20% strain. Although the PDMS-PEIE/MWCNTs-3 sensor can be stretched up to 100% strain, it has a lower linearity than the other two sensors. All of the fabricated sensors show a high repeatability since all readings have repeatable stretching and relaxing patterns during the cyclic tensile tests (Figure 10). Additionally, we tested a single PDMS-PEIE/MWNTs-1 for three different tensile speed with a total cycle of 3,000 cycles (Supplementary Figure S6). We found that the PDMS-PEIE/MWNTs-1 shows a stable performance since the curve shows a repeatable pattern at every tensile speed.

**FIGURE 11:**
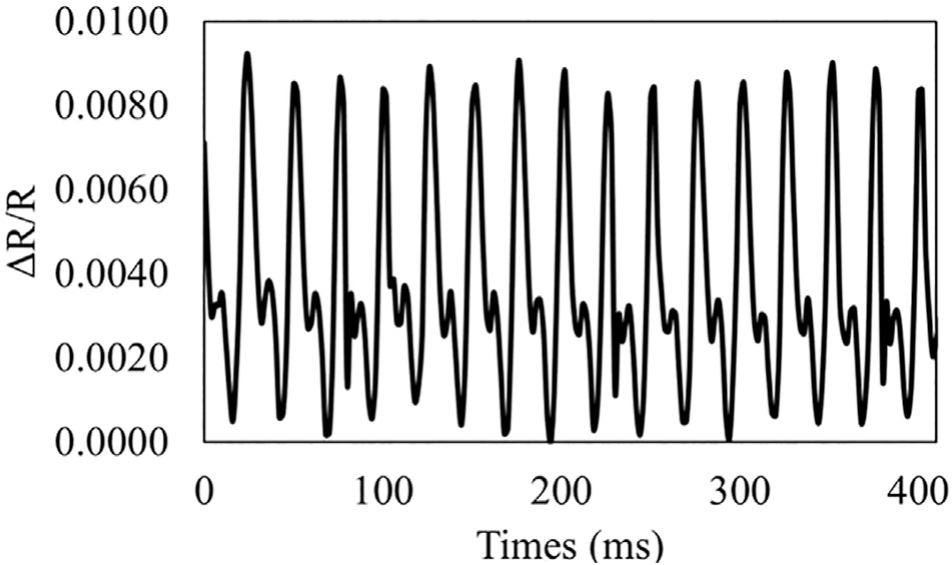
Response of a PDMS-PEIE/MWCNTs-1 stretchable sensor to the vibration at 40 Hz.

We also assessed the dynamic performance of PDMS-PEIE/MWCNTs-1 using ET139 by Labworks, Inc. (Supplementary see Figure S7 for the experimental details). PDMS-PEIE/MWCNTs-1 shows a stable performance when the sensor is subjected to a vibration frequency of 40 Hz (period of 25 ms), (Figure 11). Based on this dynamic test, we can conclude that the PDMS-PEIE/MWCNTs-1 has a responsivity up to 25 ms. Table 1 summarizes our results. By implementing our novel DIY strategy to fabricate a stretchable strain sensor, the sensitivity of the PDMS-MWCNTs-based sensor is improved.

**TABLE 1:** Comparison among the strain sensors fabricated using CNT and silicone elastomer as the stretchable electrodes.

Material	Fabrication method	Stretchability	Gauge factor	Number of cycle	Response	References
PDMS-PEIE/MWCNTs-1	Brushing of CNT powder on the elastomer surface	Up to 100%	6.2–8.2	50	25 ms	This work
PDMS-PEIE/MWCNTs-2	Brushing of CNT powder on the elastomer surface	Less noise up to 50%	19.8–27.6	50	25 ms	This work
PDMS-PEIE/MWCNTs-3	Brushing of CNT powder on the elastomer surface	High linearity up to 20%	6–10 (at 0–20% strain)	50	25 ms	This work
Ecoflex—Carbon Black	Mixed material (composite-Ecoflex and carbon black)	500%	1.62–3.37	10100	Stable at tensile speed of 10 mm/s	Shintake et al. (2018)
Ecoflex-CNT	Mixed material (composite-Ecoflex and CNT)	500%	1.75	2000	∼332 ms	Amjadi et al. (2015)
PDMS-CNT	Coating	100%	1.8	20	1000 ms	Jin et al. (2020)
Plasticized PVC-CNT	Mixing of CNT with a substrate and sandwiching electrodes with an elastomer	100%	1.16	3,000	132 ms	Dong et al. (2021)

**FIGURE 12:**
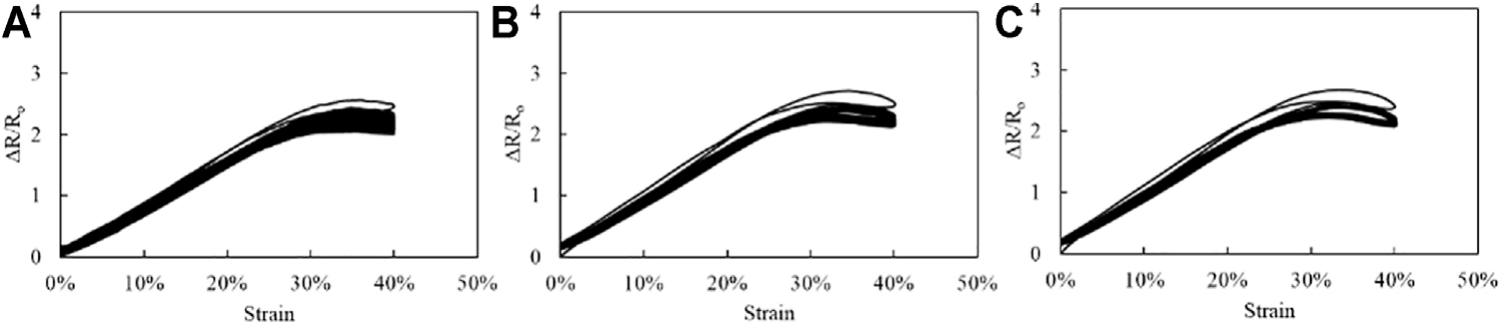
Stretchable strain sensor (PDMS-PEIE/MWCNTs-1) characteristics after the lamination process using an Ecoflex membrane at a tensile test speed of **(A)** 7 mm/s **(B)** 21 mm/s, and **(C)** 30 mm/s.

After the lamination process using exoflex thin membrane, we also check the quality of the stretchable sensor. Figure 12 shows the quality of the laminated stretchable strain sensor. Comparing with the naked stretchable strain sensor characteristics (Figure 10A), the lamination process does not affect the quality of the stretchable strain sensor (Figure 12).

### Stretchable Sensor Application for Detecting Object Movement

**FIGURE 13:**
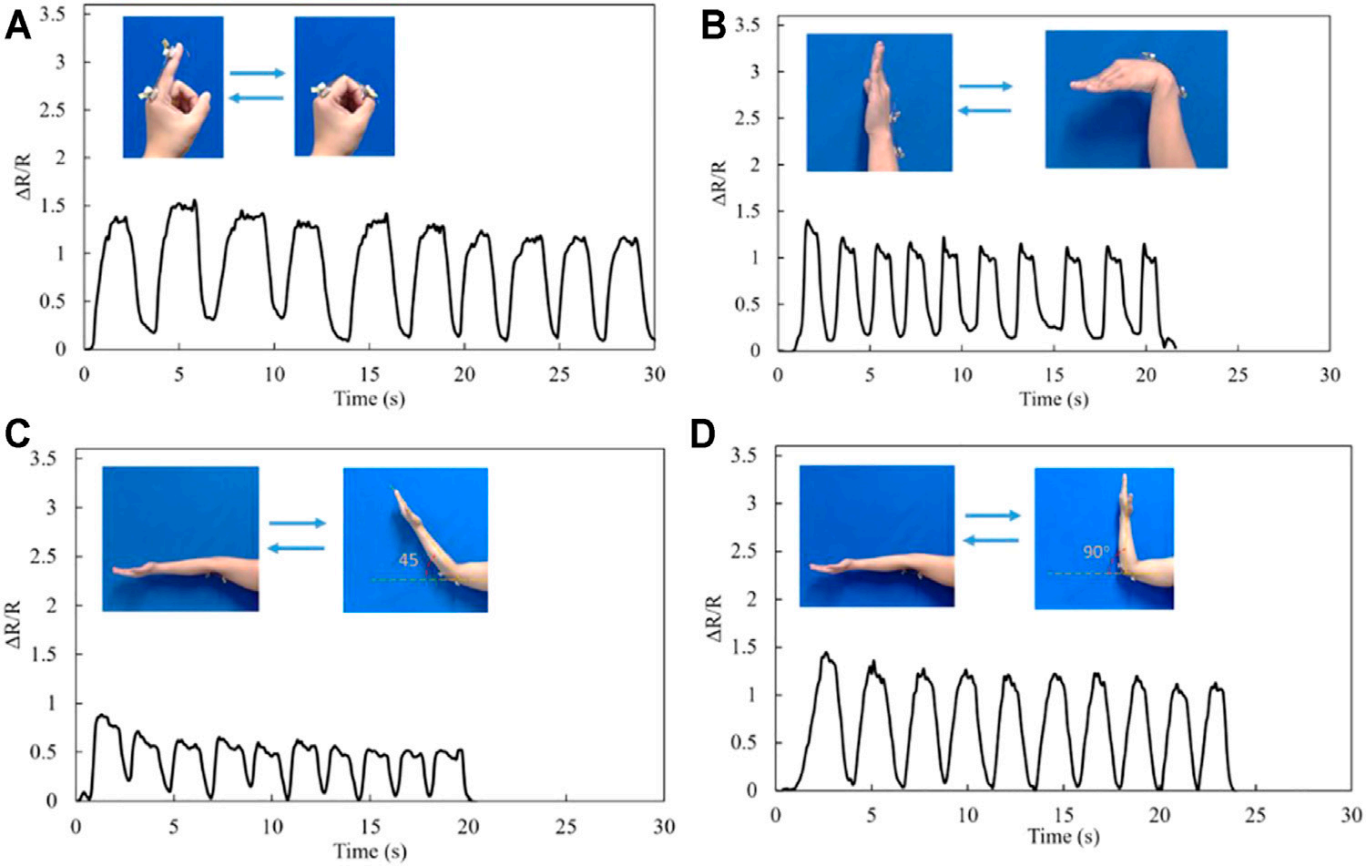
Response patterns of the PDMS-PEIE/MWCNTs-1 sensor fixed on **(A)** index finger, **(B)** wrist, **(C)** 45-degree movement of the elbow, and **(D)** 90-degree movement of the elbow.

In this section, we specifically investigated the sensitivity, and the strain range of the PDMS-PEIE/MWCNTs-1 to monitor human movement since the PDMS-PEIE/MWCNTs-1 shows stable reading, lower noise than PEIE/MWCNTs-2, and has a potential to be stretched up to 100% with low noise in the sensor reading. The response of the strain sensor increases when the index finger is bent from its natural position to the fully rolled position (Figure 13A). When the index finger moved cyclically from the natural position to the rolled position several times, the sensor reading value changes according to the movement. When the movement is terminated, the sensor value returns to its original state. Similarly, the stretchable strain sensor can detect other joint bending movements such as the wrist (Figure 13B). In addition, the response intensity of the stretchable strain sensor varies when the elbow is bent at different angles (Figures 13C,D). A larger bending angle induces a higher response in the stretchable sensor (Figure 13D).

## Discussion

The electromechanical properties of the sensor (Figure 10) confirm that each MWCNTs powder has its own strengths and weaknesses characteristics when fabricated using a brushing method. We realize that the characteristic of our stretchable sensor is affected by the MWCNTs powder agglomerates. The smaller the agglomerates of the powder can produce less noise in the sensor reading. In our case the smaller agglomerates were found in MWCNTs-1. The dispersion of the MWCNTs particles in nanometric scale can strengthen the network connection between MWCNTs particle which also realize lower noise at higher strain value of stretchable sensor. This strong and uniform dispersion of the MWCNTs powder is found in MWCNTs-1 (Figure 9a1 and 9a2). This phenomenon makes PDMS-PEIE/MWCNTs-1 has a high potential for future development of stretchable strain sensor.

On the other hand, MWCNTs-3 shows a high nonlinearity and lower noise reading of the sensor. The high nonlinearity is highly affected by the structure of the MWCNTs-3 which looks stiffer than the other MWCNTs and the less density of the MWCNTs network structure. These two combinations of less density of network structure problem and stiff structure of MWCNTs particle can cause a degradation of sensor sensitivity when the sensor is at high strain condition.

In MWCNTs-2 case, the micro metric image (Figure 9B) shows that there are more agglomerates than MWCNTs-1 and MWCNTs-3. These agglomerates make the MWCNTs-2 has higher sensitivity and noise of the sensor reading at the same time. When the sensor is integrated to the system, a filtering algorithm (e.g., moving average filter or exponential filter) can remove the noise in the sensor reading of MWCNTs-2. However, in some cases, when a low-cost microcontroller is used, the filter algorithm application can cause a delay in the sensor reading. Since more terrible noise was found at a higher strain of PDMS-PEIE/MWCNTs-2 (Supplementary Figure S5B) than PDMS-PEIE/MWCNTs-1 and PDMS-PEIE/MWCNTs-3, PDMS-PEIE/MWCNTs-2 is more suitable for sensing a strain approximately between 0 and 50%.

By reviewing the strength and weakness of each type of powder when it is used as stretchable sensor, we found that PDMS-PEIE/MWCNTs-1 shows a good performance in terms of linearity and repeatability in lower and higher strain sensing areas. Hence, we recommend fabricating a combination of PDMS-PEIE and MWCNTs-1 *via* the brushing method for low-cost strain sensing projects (e.g., human movement detection) and wearable sensor such as smart gloves, which can detect finger movements.

In the future applications, this stretchable sensor can be used as a motion detection in soft actuator, soft bio inspired robot and human-soft robot interaction project. The flexibility of the sensor and the simple fabrication makes the stretchable sensor easily to be integrated to any device.

In this current research we integrate the stretchable sensor with a low-cost micro controller to detect finger movement. The principle is by detecting a higher or lower response of the stretchable strain sensor (due to the different movement angles shown in Figures 13C,D) and mapping the value of the sensor response through a simple transfer function to control a virtual hand. We prepared a virtual hand model using Blender (opensource software from blender.org). Blender receives the mapped signal from the strain sensor attached to the glove and mimics the human hand movement. On the instrumental side (wearable device), Supplementary Figure S8 shows the glove used to detect the hand movement and the electric instrument. In this stretchable strain sensor simulation, we used economical DIY equipment including Arduino Uno as the signal receiver and signal processor from the stretchable sensor. The electric circuit consisting of 1-MΩ resistors (Supplementary Figure S8) works as a simple voltage divider so that the analog signal can be read using Arduino Uno. Moving the glove (Supplementary Figure S8) induces a difference in the resistance of the system. This resistance change due to the hand-glove finger movement influences the voltage signal read by the analog pin in Arduino. Then the signal from the analog pin is further mapped and sent to the personal computer USB port. Finally, Blender software captures the hand movement simulation. Supplementary Figure S9 shows the detail of the electric connection circuit and peripheral of the wearable device.

To improve the movement accuracy of the wearable device (Supplementary Figure S8), a simple calibration algorithm was used to detects the maximum and minimum reading values of the sensor. This simple calibration algorithm is necessary since the movement range of every finger differs by person. A person’s hand size affects the value range of the sensor readings. Then the maximum and minimum range are further mapped to vector values that the Blender software can understand.

**FIGURE 14:**
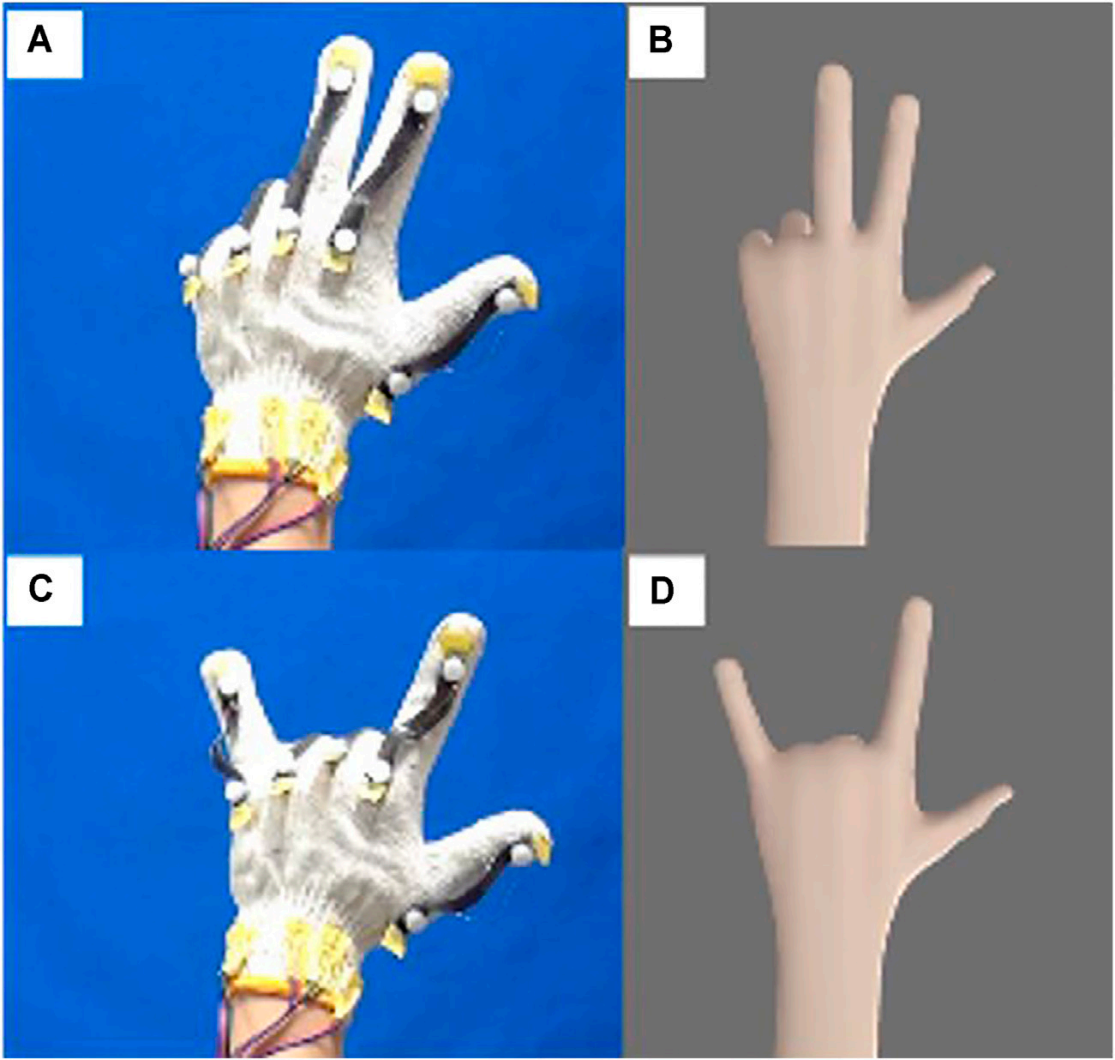
**(B and D)** Control simulation of a virtual hand using **(A and C)** a stretchable strain sensor attached to a cotton hand glove.

Figure 14 overviews the demonstration of this stretchable strain sensor attached to a cotton glove to control a virtual hand. (Supplementary Movie S2 shows a full demonstration of the control simulation, and Supplementary Figure S10 depict the strain possibility experienced by the stretchable sensor due to the finger movement). All of the materials, software, and peripherals are open-source and available in the marketplace. For instance, Sylgard 184 is commercially available with the price of $120 for 500 g, PEIE is around $100 for 100 g, MWCNTs ranges from $63 to $144 for one package. The price depends on the diameter of the MWNNTs, and one package can fabricate at least 100 samples sensors. The DIY-Kit x-y-z machine tools (SainSmart Genmitsu CNC Router 3018-PRO) is around $250, the portable enclosure glovebox (As One 3-116-01 SM-1) is around $390, while Ecoflex is around $40 per kilogram. Six nylon paintbrushes cost around $5, and the DIY-Kit Arduino is around $11. Hence, these materials and technologies are accessible and affordable for researchers studying soft robotics and stretchable sensors. Consequently, we expect that our DIY approach, which can simply fabricate an elastomer strain sensor using MWCNTs powder for a wearable sensor, can accelerate the growth of soft robotics research innovation.

Inconclusion, herein we fabricated the first carbon powder-based stretchable strain sensor produced *via* a DIY-approach and an automatic brushing machine. We highly recommend PDMS-PEIE with the composition of 3.2 wt% curing agent and 0.11 wt% PEIE for the stretchable elastomer. PDMS-PEIE is 50% more stretchable than that previously reported PDMS 30-1 (Wiranata et al., 2021) and realizes a 63% stickier elastomer. Stretchability is essential to cover a wide sensing range. Furthermore, a highly sticky surface improves the physical bonding quality between the elastomer surface and the MWCNTs powder. Strong physical binding between MWCNTs and the elastomer surface enhances the electrode conductivity (Wiranata et al., 2021) and reduces the risk of self-delamination of MWCNTs powder from the elastomer surface.

We also found that material selection for the electrode powder affects the sensor quality. Each carbon powder has its own strengths and weaknesses. MWCNTs-1 has a relatively better performance in terms of linearity and repeatability. The combination of MWCNTs-1 and PDMS-PEIE has a potential for higher strain sensing. We recommend using PDMS-PEIE/MWCNTs-1 strain sensor fabricated *via* a brushing machine for wearable devices because our demonstration shows that this material combination has a high reliability and repeatability.

## Data Availability

The original contributions presented in the study are included in the article/Supplementary Materials; further inquiries can be directed to the corresponding authors.
