# Supplementary material for: A DIY Fabrication Approach of Stretchable Sensors Using Carbon Nano Tube Powder for Wearable Device
Source: Front Robot AI. 2021 Nov 11;8:773056. doi: 10.3389/frobt.2021.773056 (PMC8632443; doi:10.3389/frobt.2021.773056)
Supplement: Supplementary file 3 [file DataSheet1.docx]

Supplementary Material


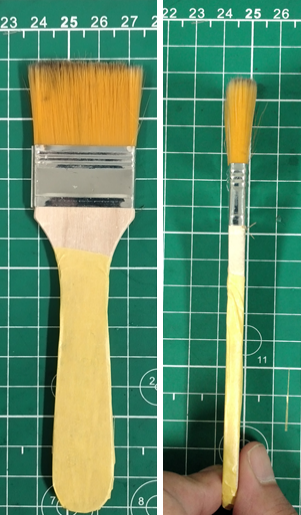


**Figure S1.** Wooden brush with synthetic nylon hair.

**Figure S1** shows the paintbrush used for the automatic brushing machine. The handle and brush are made of wood and synthetic nylon bristles, respectively. Six of these paintbrushes cost about $5.


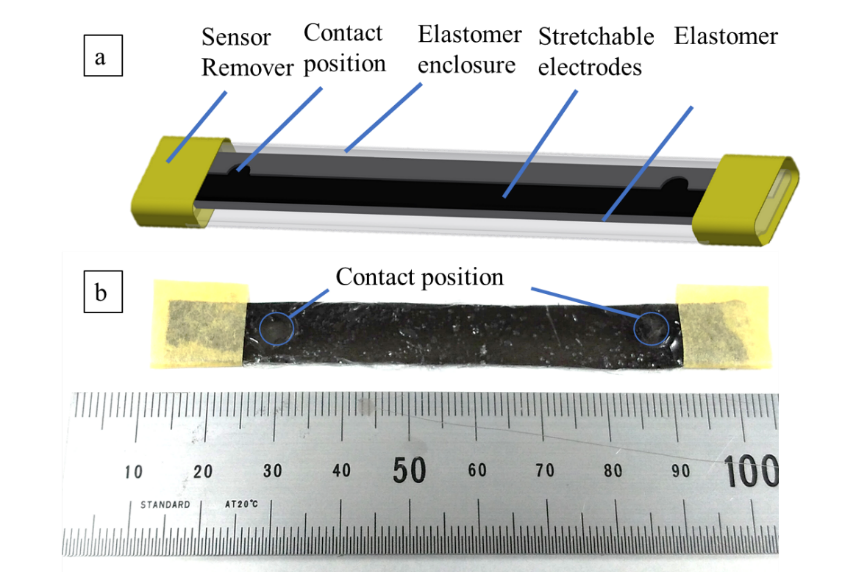


**Figure S2.** Sensor final structure after the lamination process.

To enhance the compatibility of the CNT powdered-based stretchable sensor, we performed lamination by wrapping the stretchable sensor with a thin Ecoflex membrane. **Figure S2b** shows the final structure of the stretchable sensor. The yellow tape is for handling ease when attaching or removing the sensor from the wearable device.


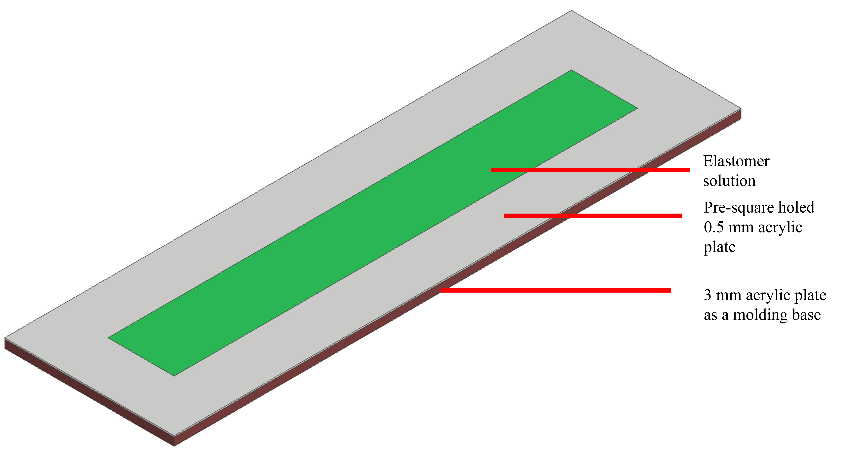


**Figure S3.** Molding shape for the loop tack test of the elastomer.

**Figure S3** shows the molding shape for the loop tack test. The molding method require a 3 mm acrylic plate as a base and 0.5 mm acrylic as a mold shaper. then we create a square hole with the size of 2.5 ×175mm on the 0.5 mm acrylic and finally we adhered this pre-holed 0.5mm acrylic to the base 3 mm acrylic plate using acrylic adhesive. then the molding methods begin with pouring the elastomer solution on to the molding.


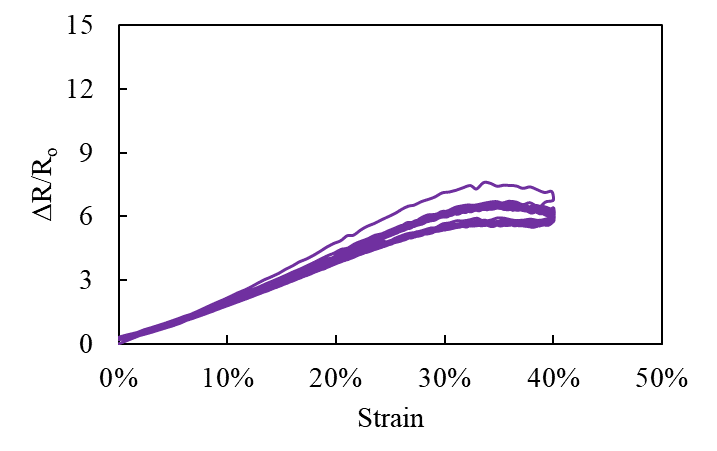


**Figure S4.** PDMS-PEIE-2/MWCNTs-2 stretchable sensor characteristics at a tensile speed of 7mm/s.


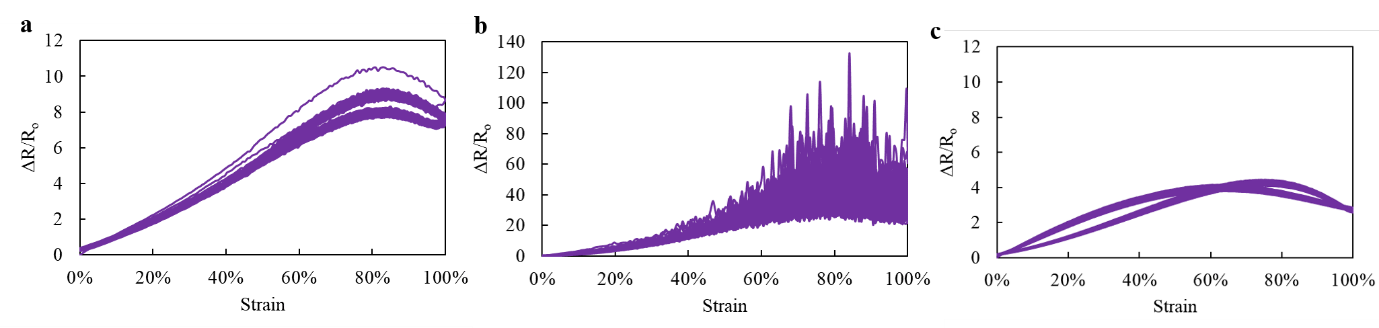


**Figure S5.** Sensor characteristics at a higher strain (a. PDMS-PEIE-2/MWCNTs-1, b. PDMS-PEIE-2/MWCNTs-2, c. PDMS-PEIE-2/MWCNTs-3)

**Figure S4** shows the characteristics of the PDMS-PEIE-2/MWCNTs-2 at a tensile speed of 7 mm/s. The sensor readings show noise, which becomes worse as the sensor is pulled at a strain of 100% (**Fig. S5b). Figure S5a** shows that the PDMS-PEIE-2/MWCNTs-1 has the potential to be stretched up to 100% with less noise and that the sensor can maintain the gauge factor until reaching a strain of around 80%. PDMS-PEIE-2/MWCNTs-3 can also sense strain up to 100% (**Fig. S5c**). However, it has a lower gauge factor than the other two sensors (**Figs. S5a** and **S5b**).


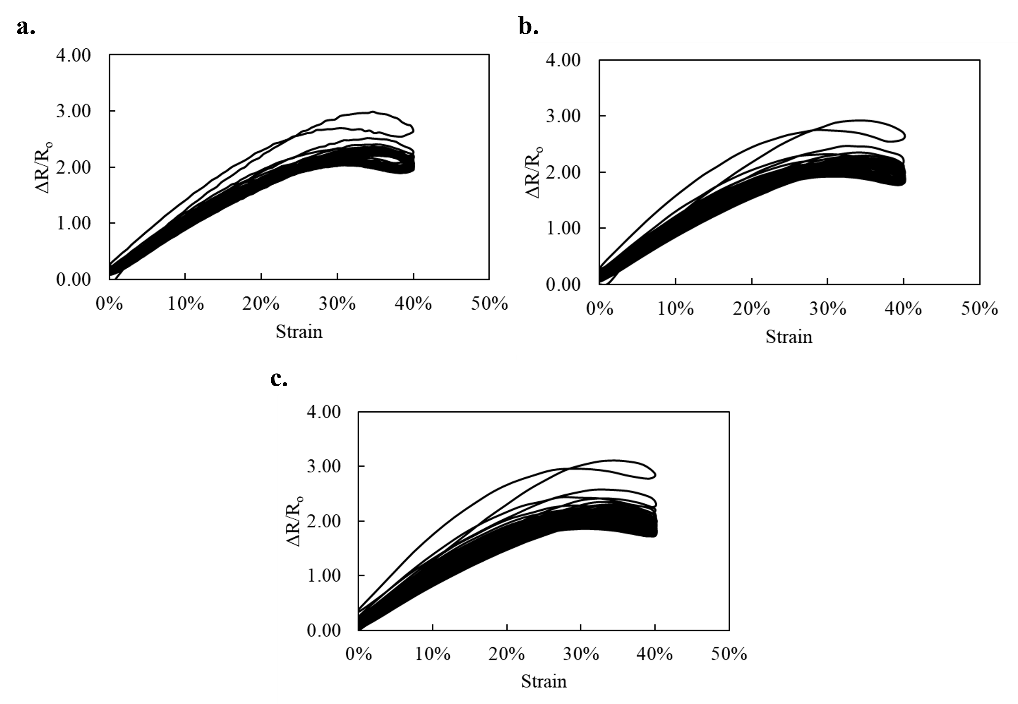


**Figure S6.** PDMS-PEIE/MWNTs-1 tested for several tensile speed for 1000 cycle in each test. a. tensile speed of 7mm/s, b. tensile speed of 21 mm/s, c. tensile speed of 30mm/s

Additionally, we also tested a stretchable sensor of PDMS-PEIE/MWNTs-1 through several different tensile speeds of 7mm/s, 21mm/s and 30mm/s, as presented in **Figure S6.** Each tensile test was a 1000 cyclic tensile test. In this additional investigation, a single PDMS-PEIE/MWNTs-1 went through 3000 cycles of stretch and relaxed test. The converged curve shown in figure S6 indicates that the sensor is reliable.


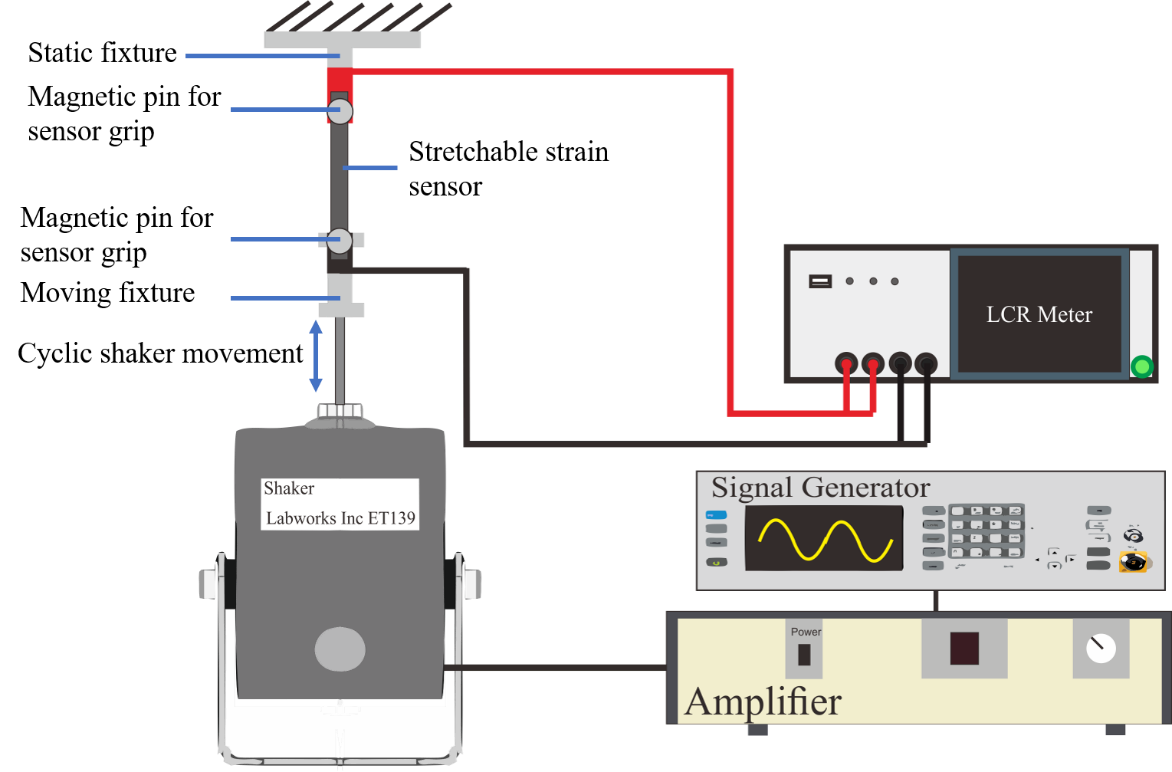


**Figure S7**. Sensor assessment for the vibration detection experimental setup.

**Figure S7** shows the equipment used to assess the sensor when subjected to different vibration frequencies. In principle, a magnetic pin connects the sensor to the shaker. We controlled the shaker frequency using a signal generator. The signal from the signal generator is amplified using an amplifier before reaching the shaker. Finally, the shaker moves cyclically with a specific frequency according to the signal generator setting. All the signal responses are recorded using an LCR meter.


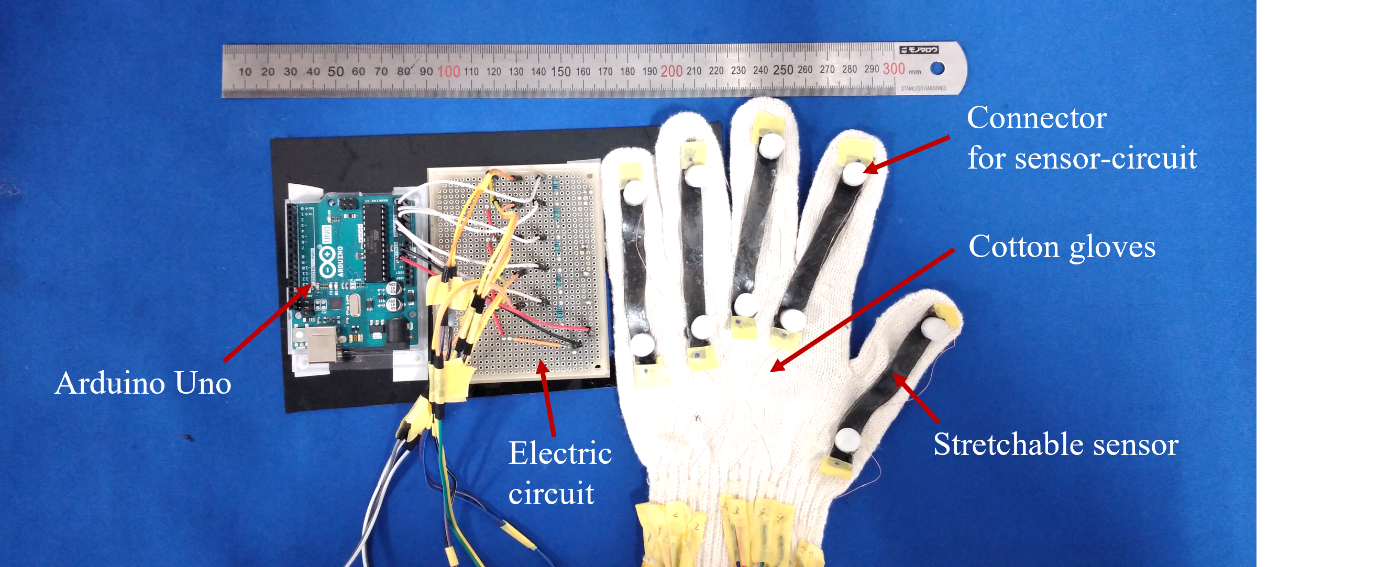


**Figure S8.** Wearable device arrangement for simulating and mimicking human hand movement.

We connected the stretchable sensor-cotton glove system with a low-cost microcontroller (Arduino) for the wearable device (**Fig. S8**). In this prototype, we used the device to control a virtual hand (**Movie S2**)**. Figure S9** schematically depicts of the electric circuit**.** The 1-MΩ resistor works as a simple voltage divider, and the analog signal from the voltage divider-cotton gloves system is transmitted to the Arduino. Finally, the signal is received to control the virtual hand displayed on the personal computer.

**
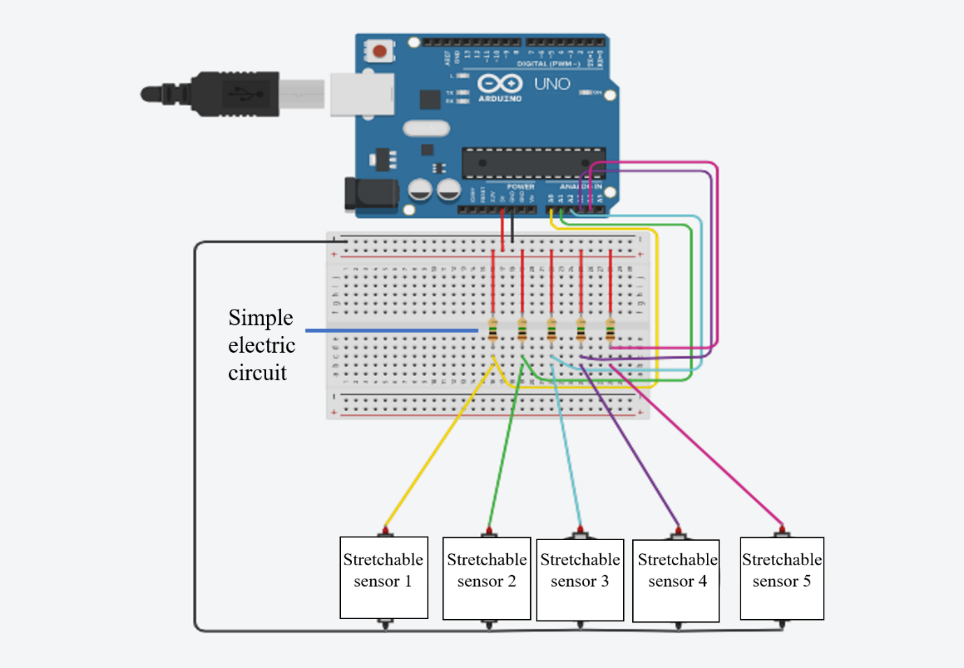
**

**Figure S9.** Detailed electric circuit for the stretchable strain sensor reader and signal processor.


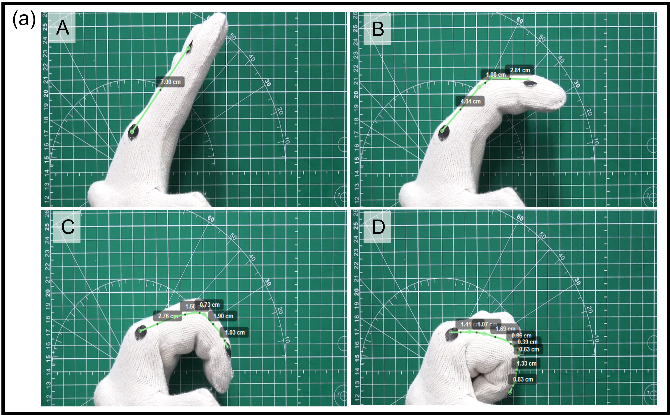

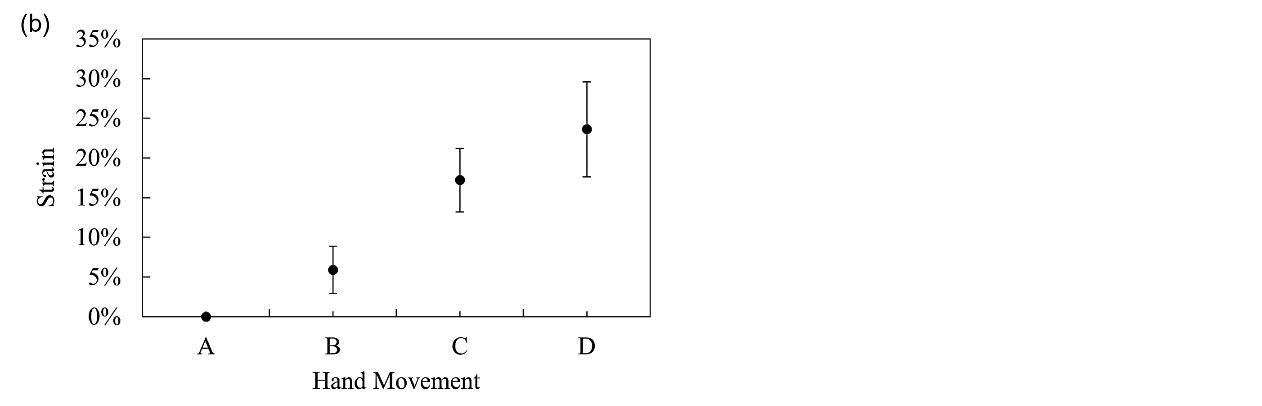


**Figure S10.** Possibility strain vs hand movement. (a). hand movement code (A,B,C and D), (b). Hand movement code vs possibility strain due to the hand movement.

In order to investigate the possibility strain vs the movement of the finger, we additionally create a simple test involving three respondent with different hand size. Each test was conducted at least three times. The result of the test is shown in Figure S9. When our hands are clenched as presented in **Figure S9(a)D,** our stretchable sensor may strained approximately around 25%.
